# Editorial

**Published:** 2014-04-26

**Authors:** Nikhil Marwah

## Abstract

Sometimes the most treasured gift is usually the fruit of the most laborious efforts. Ever since we became eligible to apply for PubMed, in the beginning of 2011, we were steadfast in moving toward the indexing goal.

## IJCPD – Listed in PubMed … New horizons … New challenges

Sometimes the most treasured gift is usually the fruit of the most laborious efforts. Ever since we became eligible to apply for PubMed, in the beginning of 2011, we were steadfast in moving toward the indexing goal. There were times during this entire tenure when our efforts did not bear the desired results and the target seemed a long way off. But kudos the entire team of IJCPD who withstood the text of time and finally got the journal its well-earned recognition. It gives me immense pride and pleasure to inform all the readers that International Journal of Clinical Pediatric Dentistry is now listed with PubMed.

I whole heartedly express my gratitude to the technical team of IJCPD led by Ms. Chetna Malhotra without whom this would have never been possible. I would also like to place my appreciation for the erstwhile editors, editorial board members and reviewers whose efforts have made the academic content of this journal up to international standards.

Yes, we have reached our aim but the destination is still to come and the real work has just begun. The journal would now strive hard to maintain the standards of PubMed and provide all the readers with new, informative, and original Scientific content. I, on behalf of IJCPD, thank all the subscribers and authors for believing in us and look forward for your continued patronage and support.

                                           *… Next Aim … Impact Factor …*


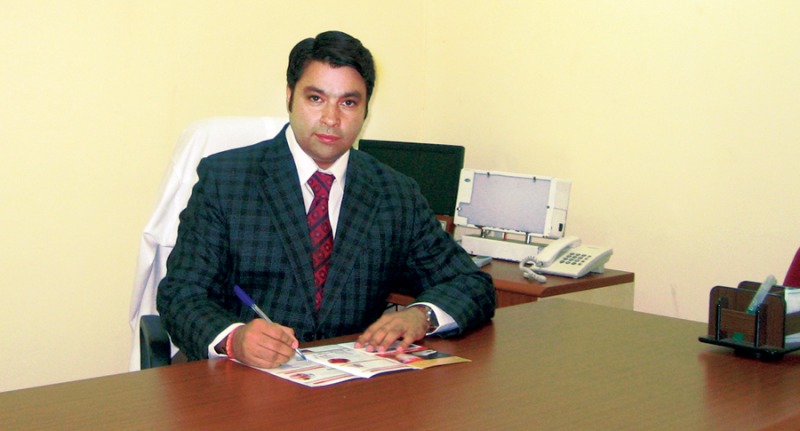
**Nikhil Marwah**
Editor-in-chief


